# TNF Family–Based Signature Predicts Prognosis, Tumor Microenvironment, and Molecular Subtypes in Bladder Carcinoma

**DOI:** 10.3389/fcell.2021.800967

**Published:** 2022-01-31

**Authors:** Huihuang Li, Siyuan Liu, Chenxuan Li, Zicheng Xiao, Jiao Hu, Cheng Zhao

**Affiliations:** ^1^ Department of Urology, Xiangya Hospital, Central South University, Changsha, China; ^2^ National Clinical Research Center for Geriatric Disorders, Xiangya Hospital, Central South University, Changsha, China; ^3^ Department of General Surgery, The Second Xiangya Hospital, Central South University, Changsha, China; ^4^ Xiangya School of Medicine, Central South University, Changsha, China

**Keywords:** bladder carcinoma, prognosis, tumor microenvironment, immunotherapy, molecular subtype

## Abstract

**Background:** Tumor necrosis factor (TNF) family members play vital roles in cancer development and antitumor immune responses. However, the expression patterns, prognostic values, and immunological characteristics of TNF members in bladder carcinoma (BLCA) remain unclear.

**Methods:** The training cohort, TCGA-BLCA, was downloaded from The Cancer Genome Atlas; another two Gene Expression Omnibus datasets (GSE13507 and GSE32894) and the Xiangya cohort (RNA-sequencing cohort collected from our hospital) were used as the external validation cohort. The least absolute shrinkage and selection operator (LASSO) algorithm and cross-validation were used to screen variables. Cox regression model and random survival forest (RSF) were used to develop the risk score, respectively. Then, we systematically correlated the TNF risk score with the tumor microenvironment (TME) cell infiltration, molecular subtypes of BLCA, and the potential value for predicting the efficacy of immunotherapy.

**Results:** We developed two TNF-based patterns, named TNF cluster 1 and TNF cluster 2. TNF cluster 1 exhibited poorer survival outcome and an inflamed TME characteristic compared with TNF cluster 2. We then filtered out 196 differentially expressed genes between the two TNF clusters and applied the LASSO algorithm and cross-validation to screen out 22 genes to build the risk score. For risk score, we found that RSF exhibited higher efficacy than the Cox regression model, and we chose the risk score developed by RSF for the following analysis. BLCA patients in the higher risk score group showed significantly poorer survival outcomes. Moreover, these results could be validated in the external validation cohorts, including the GSE13507, GSE32894, and Xiangya cohorts. Then, we systematically correlated the risk score with TME cell infiltration and found that it was positively correlated with the infiltration of a majority of immune cells. Also, a higher risk score indicated a basal subtype of BLCA. Notably, the relationship between risk score, TME cell infiltration, and molecular subtypes could be validated in the Xiangya cohort.

**Conclusion:** We developed and validated a robust TNF-based risk score, which could predict prognostic outcomes, TME, and molecular subtypes of BLCA. However, the value of risk score predicting the efficacy of immunotherapy needs further research.

## Introduction

Bladder carcinoma (BLCA) is the 11th most common carcinomas globally, with nearly 550,000 new cases and 200,000 new deaths every year (Bray et al., 2018). BLCA can be divided into muscle-invasive bladder carcinoma (MIBC) and non–muscle-invasive bladder carcinoma (NMIBC) based on whether the tumors invade the detrusor muscle (Sanli et al., 2017). MIBCs account for approximately 20% of newly diagnosed BLCA, and 15% to 20% of NMIBC cases tend to progress to MIBC, which is a more aggressive form of cancer with extremely poor survival outcomes (Patel et al., 2020). Moreover, 10% of diagnosed BLCA cases are spread beyond the bladder, with only 5% to 30% 5-year overall survival (OS) rate (Siegel et al., 2018). Therefore, discovering specific prognostic methods for the OS of BLCA is needed to choose the most suitable treatment options for distinct subsets of BLCA patients.

Because of the poor prognosis, the US Food and Drug Administration has approved five immune checkpoint inhibitors (ICIs) for the treatment of metastatic BLCA (Patel et al., 2020). As a result, the immune component of the tumor microenvironment (TME) has reinvigorated the interest of researchers. ICIs can reinvigorate anticancer immune responses by targeting inhibitory receptors on T cells, and they have achieved remarkable success in treating different types of carcinomas (Havel et al., 2019). However, only a subset of patients benefits from ICI treatment. Engaging costimulatory receptors is another promising way for promoting T-cell responsiveness (Tran and Theodorescu, 2020). Tumor necrosis factor (TNF) family members, which contain 29 TNF receptors (TNFRSF) and 19 TNF ligands (TNFSF), play a vital role in the immune system through either coinhibited or costimulated ways (Dostert et al., 2019). Therefore, regulating the comprehensive interactions between TNF members is a promising carcinoma treatment option. However, the expression patterns, prognostic values, and immunological characteristics of TNF family members in BLCA remain unclear. In this study, we conducted a comprehensive analysis of TNF family members regarding prognosis, TME, and molecular subtypes of BLCA.

## Materials and Methods

### Sources of BLCA Datasets and Preprocessing

We enrolled 932 BLCA cases from three public datasets and an RNA-sequencing (RNA-seq) cohort collected from Xiangya hospital. For The Cancer Genome Atlas (TCGA) database, the fragments per kilobase per million mapped fragments (FPKM) and the count value of 408 BLCA samples were downloaded from Genomic Data Commons (GDC, https://portal.gdc.cancer.gov/) (Colaprico et al., 2016), and then we transformed the FPKM value into transcripts per kilobase million value. After filtering duplicated patients and patients lacking full follow-up information, 403 patients from TCGA-BLCA were finally enrolled. Another two Gene Expression Omnibus (GEO) (https://www.ncbi.nlm.nih.gov/geo/) datasets with clinical and survival information were also enrolled. There are 308 cases in GSE32894 (Sjödahl et al., 2012), and we excluded 84 cases without survival information. There are 188 tumor cases and 67 normal cases in GSE13507 (Lee et al., 2010), and we excluded 23 tumor cases without survival information. Finally, for GEO databases, we included 224 cases from GSE32894 and 165 cases from GSE13507. As our previous study reported (Hu et al., 2021), we developed an RNA-seq cohort based on Xiangya hospital (Xiangya cohort) and uploaded it on the GEO database (GSE188715), which included 56 patients with survival information. The clinical information of these four datasets is summarized in Supplementary Table S1.

### Consensus Clustering

Forty-seven TNF family genes, including 18 TNFSF and 29 TNFRSF genes, were collected from a previous study (Supplementary Table S2) (Zhang et al., 2020). We excluded TNFRSF6B because of its zero expression in TCGA-BLCA and included 46 TNF family genes for further analysis. We applied the consensus clustering algorithm (maxK = 5, reps = 100, pItem = 0.8, distance = “manhattan,” clusterAlg = “pam”) implemented in the “ConsensuClusterPlus” R package to identify distinct TNF-related patterns based on these 46 TNF family genes (Wilkerson and Hayes, 2010).

### Depicting Tumor Immune Microenvironment of BLCA

In summary, the tumor immune microenvironment (TIME) of BLCA in this study included the activation of the anti–cancer-immunity cycle, infiltration of immune cells, and expression of ICI genes, effector genes of immune cells, and T cell–associated inflammatory signature (TIS). The activation levels of seven-step anti–cancer-immunity cycles were downloaded from the tracking tumor immunophenotype (TIP) (http://biocc.hrbmu.edu.cn/TIP/) (Xu et al., 2018), which is a web-based analytical platform. Then, the single-sample gene set enrichment analysis (ssGSEA) algorithm was applied to calculate individual immune cells in the TME, and the gene set for calculating is summarized in Supplementary Table S3 (Charoentong et al., 2017). In addition, we summarized 22 ICI genes, 18 TIS genes, and effector genes of immune cells, including CD8^+^ T cells, dendritic cells (DCs), macrophages, natural killer (NK) cells, and type 1 T helper (T_H_1) cells from our previous study (Supplementary Table S4) (Hu et al., 2021).

### Gene Set Variation Analysis and Molecular Subtypes of BLCA

Gene signatures that had a close association with the clinical response to immune checkpoint blockade (ICB) therapy and molecular subtypes of BLCA were summarized from the studies by Mariathasan et al. and Kamoun et al., respectively (Supplementary Table S5) (Mariathasan et al., 2018; Kamoun et al., 2020). We then performed enrichment gene set variation analysis (GSVA) based on these signatures using the “GSVA” R package (Hänzelmann et al., 2013). In addition, seven independent molecular subtype classifications were developed using the “ConsensusMIBC” and “BLCAsubtyping” R packages as our previous study described (Hu et al., 2021). Based on the classification methods reported in consensus subtype, we renamed different names of molecular subtypes into “basal” and “luminal” subtypes (Kamoun et al., 2020).

### Differentially Expressed Genes Filtering and Functional Annotation

We applied empirical Bayesian algorithm implemented in the “limma” R package to identify differentially expressed genes (DEGs). The genes with absolute log2 fold change greater than 2 and adjusted *p* < 0.01 were considered as DEGs. We then conducted Gene Ontology (GO) and Kyoto Encyclopedia of Genes and Genomes (KEGG) analyses using the “ClusterProfiler” R package.

### The Development and Validation of a Risk Score Based on DEGs

We first applied univariate Cox analysis to identify the genes with prognostic value based on the DEGs using the “survival” R package. We then selected the prognostic genes to perform the least absolute shrinkage and selection operator (LASSO) regression using the “glmnet” R package. We filtered 24 genes to develop a risk score; among these genes, IL9R and TSPAN8 were ruled out because of nonexpression in the validation cohort. Finally, 22 TNF-associated genes were enrolled, and a TNF-based risk score was developed using the “rfsrc” function implemented in the “randomForestSRC” R package. In addition, we also developed a risk score using Cox proportional hazard regression analysis implemented in the “glmnet” R package:

Risk score=∑βi∗RNAi.

We set the median value of the TNF-based risk score as the cutoff value and divided the patients into high and low TNF risk score groups. The Kaplan–Meier (K-M) method and log-rank test implemented in the “survminer” R package were used to plot the survival curves, and the timeROC function implemented in the “tROC” R package was used to assess the predictive accuracy of the risk score.

### Statistical Analysis

Pearson or Spearman correlation coefficients were applied to evaluate the correlations among the variables, and *t* test or Mann-Whitney *U* test was applied to evaluate the differences between binary groups with continuous variables. The K-M method and log-rank test were applied to plot the survival curves, and univariate Cox analysis and LASSO algorithm were applied to narrow down the candidate genes for developing risk score. The values of hazard ratio for the candidate genes were calculated by univariate Cox regression model. Both random survival forest (RSF) and Cox proportional hazard regression analysis were applied to develop the risk score, and the predictive accuracy of the TNF-based risk score was assessed using time-dependent receiver operating characteristic (ROC) analysis. Multivariate Cox regression model was chosen for calculating independent prognostic value of the TNF-based risk score. *p* < 0.05 was set as the significant criteria, and the two-sided statistical tests were applied. R software (4.0.3) was used for all analyses.

## Results

### Development of TNF-Based Patterns and Association With TIME

**FIGURE 1:**
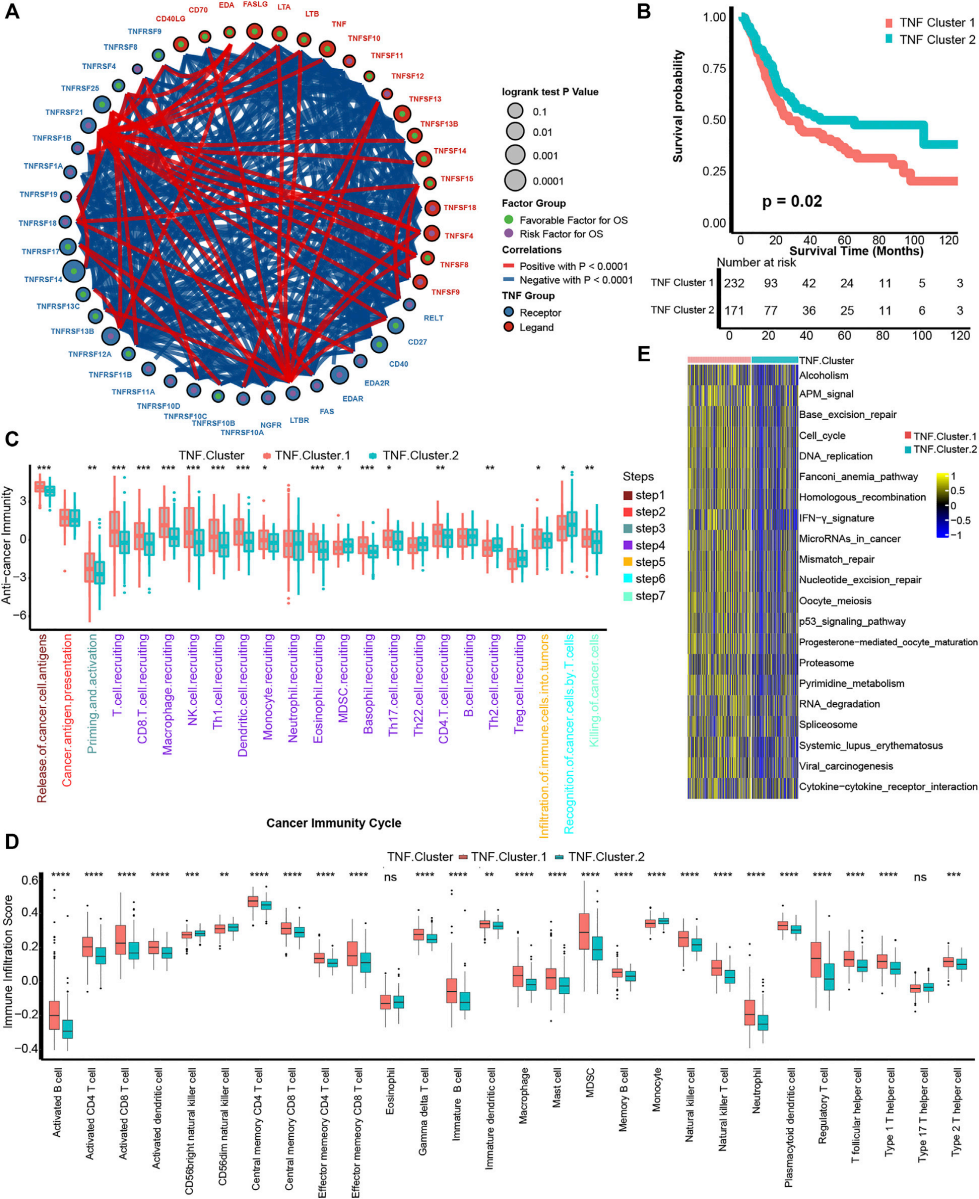
Development of TNF-based patterns and association with TIME. **(A)** The interaction between TNF family members in bladder carcinomas. The size of the circle represents the *p* value for overall survival (OS) calculated using log-rank test. The green and purple dots in the circle represent the favorable and risk factors for OS, respectively; red and blue lines represent positive and negative correlations between TNF family members, respectively. **(B)** K-M plot of OS between two TNF-based patterns; red and azure lines represent TNF clusters 1 and 2, respectively. **(C)** The different levels of anticancer immunity between two TNF-based patterns; red and azure lines represent TNF cluster 1 and 2, respectively. **(D)** The different infiltration levels of 28 immune cells in the TME using ssGSEA algorithm between two TNF-based patterns; red and azure lines represent TNF clusters 1 and 2, respectively. **(E)** The different activated levels of gene signatures associated with ICB response between two TNF-based patterns. Red and azure lines represent TNF clusters 1 and 2, respectively. *p < 0.05, **p < 0.01, ***p < 0.001, ****p < 0.0001; ns, not statistically significant.

Figure 1Ashows the comprehensive landscape of 46 TNF family genes connection, interaction, and prognostic roles. TNF family genes had a close relationship with each other, and most of the genes had prognostic roles. So, we conducted an unsupervised clustering analysis using the “ConsensuClusterPlus” R package based on these 46 TNF family genes and found that dividing the TCGA-BLCA patients into two patterns was most suitable (Supplementary Figure S1). We named these two patterns as TNF cluster 1 and TNF cluster 2 and found that TNF cluster 1 exhibited significantly poorer survival outcome compared with cluster 2 (*p* = 0.02, Figure 1B).

We then wondered if these TNF-based patterns had regulation roles in the TME. A series of stepwise actions should be activated, proceeded, and expanded to effectively kill carcinoma cells; these series of steps were named cancer-immunity cycles and consisted of seven steps (Chen and Mellman, 2013). We found that a majority of these steps were activated significantly higher in the TNF cluster 1 than cluster 2, including T-cell recruiting, CD8 T-cell recruiting, NK cell recruiting, infiltration of immune cells into tumors, and killing of cancer cells (Figure 1C). This result indicated that TNF cluster 1 might represent an inflamed TME of BLCA and could be more sensitive to ICB therapy (Gajewski, 2015; Zemek et al., 2019). We then directly figured out the infiltration levels of 28 immune cells in the TME using ssGSEA algorithm and confirmed that TNF cluster 1 could be an inflamed phenotype with higher infiltration of immune cells, including activated CD4 T cells, activated CD8 T cells, macrophages, NK cells, and T_H_1 cells (Figure 1D). Mariathasan et al. developed gene signatures that are closely associated with clinical response to ICB therapy (Mariathasan et al., 2018). As shown in Figure 1E, all these 21 gene signatures were significantly activated in TNF cluster 1, indicating that patients in TNF cluster 1 could be more sensitive to ICB therapy.

### Development of a TNF Family–Based Risk Score in the TCGA-BLCA Cohort

**FIGURE 2:**
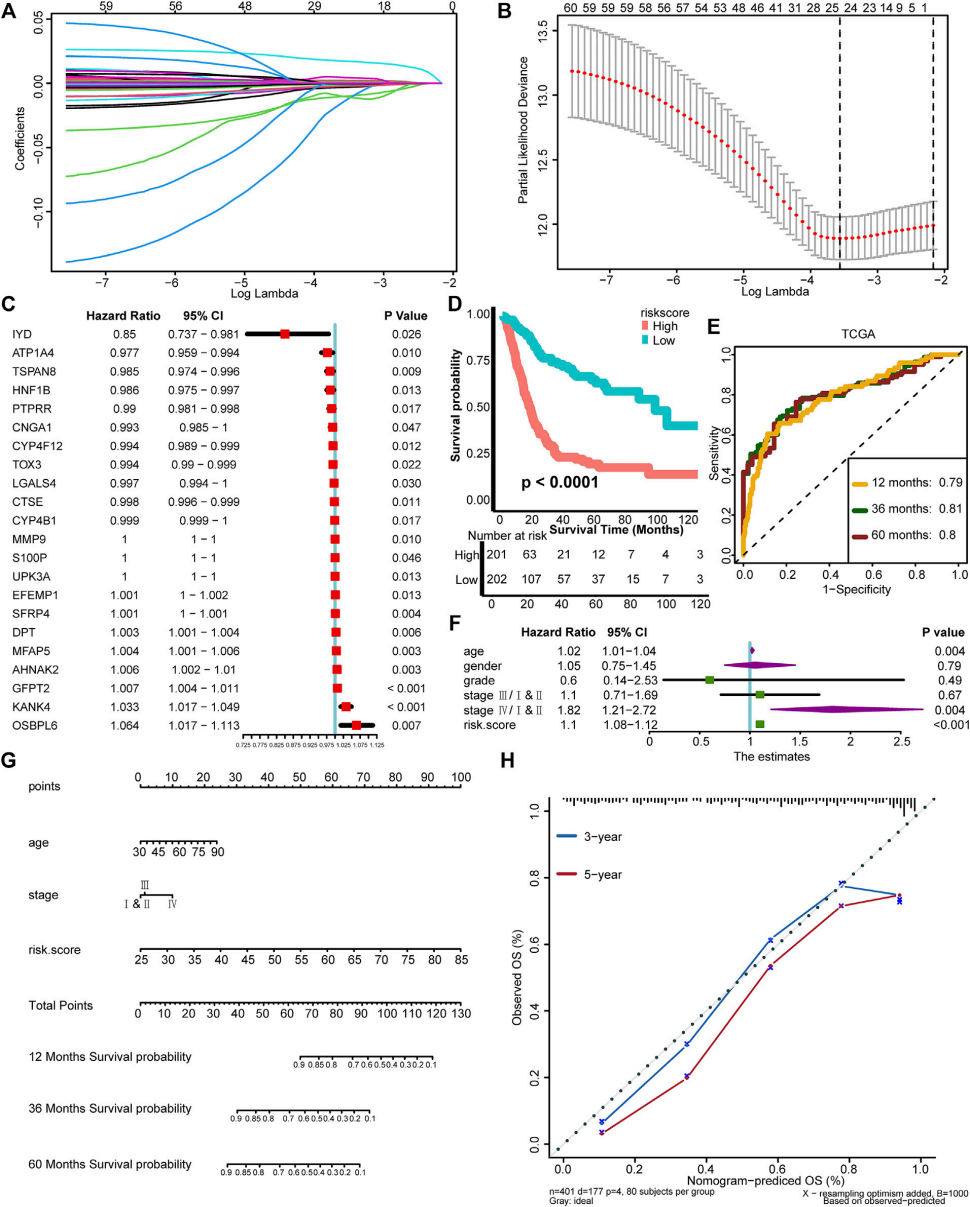
Development of a TNF family–based risk score in the TCGA-BLCA cohort. **(A)** Coeffcients of TNF-related DEGs with prognostic value are shown by lambda parameter. **(B)** Partial likelihood deviance versus log (lambda) drawn by LASSO algorithm and cross-validation. **(C)** The univariate analysis of 22 TNF-associated genes selected for developing risk score is shown in forest plots. **(D)** K-M plot of OS between TNF-based risk score groups; red and azure lines represent high and low TNF-based risk score groups, respectively. **(E)** The area under the curve (AUC) plot of TNF-based risk score in TCGA training cohort. **(F)** Forest plots of multivariate Cox analysis of TNF-based risk score combined with age, gender, tumor grade, and stage of BLCA. **(G)** Nomogram developed by using age, tumor stage, and TNF-based risk score. **(H)** Calibration curves of the nomogram.

We have depicted two TNF-based patterns and correlated them with survival outcome and TIME. In order to conduct a personalized evaluation of the role of TNF family genes in BLCA, we further developed a TNF family–based risk score. First, we filtered out 196 DEGs between TNF cluster 1 and cluster 2 using the “limma” R package (Supplementary Figures S2A, B, Supplementary Table S6). GO and KEGG analysis revealed that these 196 DEGs could be enriched in some immune-related pathways, like collagen-containing extracellular matrix and cytokine activity (Supplementary Figures S2C–F, Supplementary Table S7), which had driven us to correlate the risk score with TIME and ICB response in the next step. Among these 196 DEGs, 60 genes possessed prognostic values using univariate Cox analysis (Supplementary Table S8). We further narrowed down these genes using LASSO and 10-fold cross-validation. We identified 24 candidate genes with minimal lambda (0.11), and IL9R and TSPAN8 were ruled out because of nonexpression in the validation cohort (Figures 2A, B). Finally, 22 TNF-associated genes were enrolled, and a TNF-based risk score was developed using the “rfsrc” function implemented in the “randomForestSRC” R package. The prognostic values of these genes are shown in Figure 2C. As shown in Figure 2D, the patients with high TNF-based risk score exhibited significantly poorer survival outcomes (*p* < 0.0001), and the predictive accuracy for 12, 36, and 60 months were 0.79, 0.81, and 0.80, respectively (Figure 2E), indicating relatively high predictive accuracy. However, the accuracy of the risk score developed using Cox proportional hazard regression analysis was lower than the RSF developed risk score (Supplementary Figures S3A, B). Therefore, we chose the risk score developed using RSF for further analysis. Combined with age, gender, tumor grade, and stage of BLCA, TNF-based risk score still remained an independent risk factor (*p* < 0.001) in multivariate Cox analysis (Figure 2F). We then developed a nomogram by combining the TNF-based risk score and other clinicopathological characteristics with independent prognostic values in multivariate Cox analysis, including age and tumor stage (Figure 2G). The OS predicted by the nomogram was generally consistent with the actual OS, indicating the potentiality in the clinical application of this nomogram (Figure 2H).

### External Validation of the Risk Score

**FIGURE 3:**
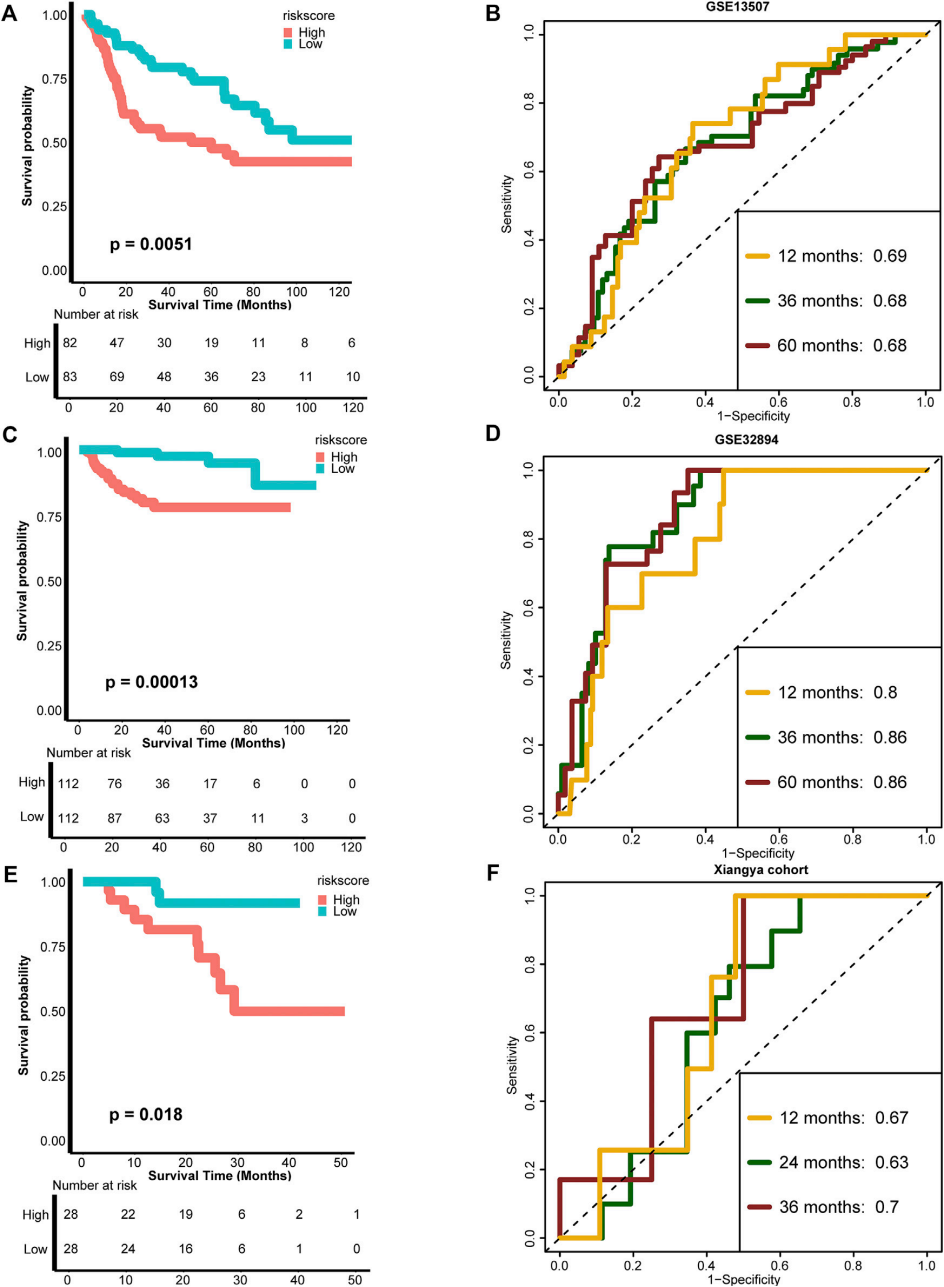
External validation of the TNF-based risk score. **(A**,**B** **)** K-M plot of OS between TNF-based risk score groups and AUC plot of the risk score in the GSE13507 validation cohort, respectively. **(C**,**D)** K-M plot of OS between TNF-based risk score groups and AUC plot of the risk score in the GSE32894 validation cohort, respectively. **(E**,**F)** K-M plot of OS between TNF-based risk score groups and AUC plot of the risk score in Xiangya validation cohort, respectively. Red and azure lines represent high and low TNF-based risk score groups, respectively.

To test the extrapolation of our risk score, we validated the risk score using external cohorts, including GSE13507, GSE32894, and Xiangya cohort. As shown in Figures 3A, B, the patients with higher risk score in GSE13507 also exhibited significantly poorer survival outcomes (*p* = 0.0051), and the predictive accuracies for 12, 36, and 60 months were 0.69, 0.68, and 0.68, respectively. For GSE32894, the survival outcomes in the high-risk group were still poorer (*p* = 0.00013), and the predictive accuracies for 12, 36, and 60 months were 0.80, 0.86, and 0.86, respectively, indicating relatively high predictive accuracy of our risk score (Figures 3C, D). The same for Xiangya cohort, the patients in the high-risk score group exhibited poorer survival outcomes (*p* = 0.018), and the predictive accuracies for 12, 24, and 36 months were 0.67, 0.63, and 0.70, respectively (Figures 3E, F). The risk score developed using Cox proportional hazard regression analysis was not satisfied as developed using RSF (Supplementary Figures S3B–H). All these results indicated that our risk score could be a robust predictive tool for OS of BLCA.

### Association Between TNF Family–Based Risk Score and TIME and ICB Response

**FIGURE 4:**
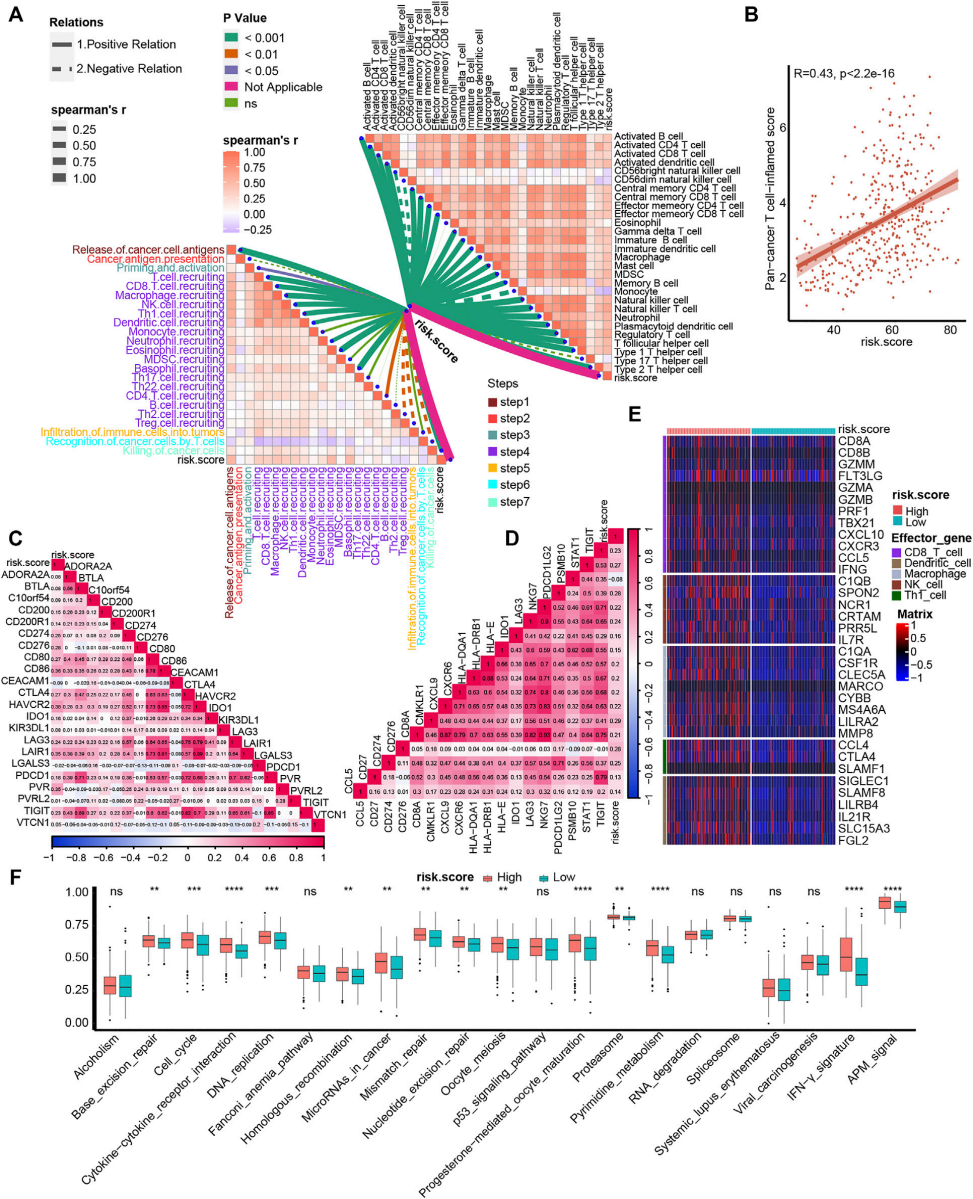
Association between TNF family–based risk score and TIME and ICB response. **(A)** The association between TNF-based risk score and cancer-immunity cycles **(left)** and immune cells in the TME **(right)**. The different types of lines represent the positive or negative relations. The different colors of the lines represent the *p* values of the relations, and the thickness of the lines represents the strength of the relations. **(B)** The association between TNF-based risk score and T cell–associated inflammatory signature (TIS) score. **(C**, **D)** The association between TNF-based risk score and immune checkpoint inhibitor (ICI) genes and TIS genes, respectively. **(E)** The different expression patterns of effector genes of immune cells between different TNF-based risk score groups. **(F)** The different activated levels of gene signatures associated with ICB response between different TNF-based risk score groups. Red and azure lines represent high and low TNF-based risk score, respectively. **p < 0.01, ***p < 0.001, ****p < 0.0001; ns, not statistically significant.

One of the main obstacles in understanding and treating carcinoma is the high heterogeneity of the TME (Duan et al., 2020). So, we correlated our TNF family–based risk score with TIME and detected its potential biomarker role for ICB response. For cancer-immunity cycles, TNF-based risk score was significantly positively correlated with the majority of these seven steps, including T-cell recruiting, CD8 T-cell recruiting, macrophage recruiting, T_H_1 cell recruiting, and killing of cancer cells (Figure 4A, left, Supplementary Table S9). Moreover, the risk score was significantly positively associated with immune cells in the TME, such as activated CD4 and CD8 T cells, macrophages, and T_H_1 cells (Figure 4A, right, Supplementary Table S9). Unlike TNF-based patterns, our TNF-based risk score could evaluate the patients’ individual TME phenotypes and guide treatment options. We further found that the risk score was positively correlated with the TIS score (Figure 4B). As shown in Figures 4C, D, the risk score was positively correlated with most of the ICI genes and TIS genes (Supplementary Table S10). The patients in the high-risk score group expressed higher effector genes of immune cells, including CD8^+^ T cells, DCs, macrophages, NK cells, and T_H_1 cells (Figure 4E). We further evaluated the level of ICB response–associated pathways between high- and low-risk score groups and found that almost all these pathways were activated in the high-risk score group. These results indicated that patients with higher risk score represented an inflamed phenotype and might be more sensitive to ICB therapy.

### TNF Family–Based Risk Score Stratified Molecular Subtypes of BLCA

**FIGURE 5:**
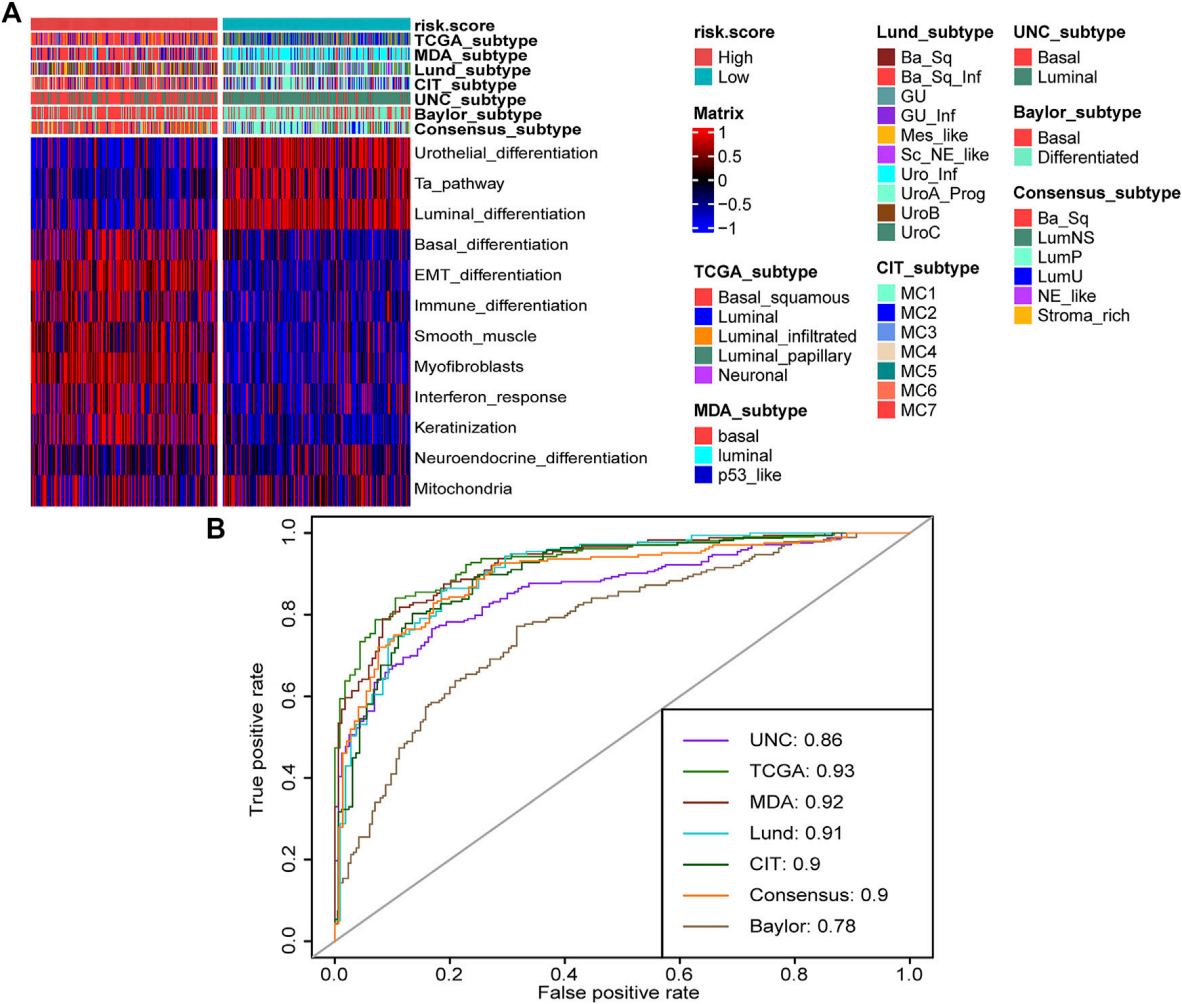
TNF family–based risk score stratified molecular subtypes of BLCA in TCGA cohort. **(A)** The heat map of different TNF-based risk score groups, seven molecular subtype classifications, and bladder cancer–associated signatures in BLCA. Activated or inhibited pathways are marked as red or blue, respectively. **(B)** AUC plot of the risk score for predicting seven molecular subtype classifications in BLCA.

Molecular subtypes were extensively researched topics that could predict the prognosis and treatment response of BLCA (Warrick et al., 2019). There are seven reported BLCA molecular classifications (Kamoun et al., 2020). However, the different numbers, sizes, and names of molecular subtypes, as well as the complicated detected methods, impeded their clinical application. In this study, we found that the high-risk score group represented the basal subtype of BLCA across seven molecular classifications, including TCGA subtype (Robertson et al., 2017), MDAnderson Cancer Center (MDA) subtype (Choi et al., 2014), Lund subtype (Marzouka et al., 2018), Cartes d’Identité des Tumeurs-Curie (CIT) subtype (Rebouissou et al., 2014), University of North Carolina (UNC) subtype (Damrauer et al., 2014), Baylor subtype (Mo et al., 2018), and consensus subtype (Kamoun et al., 2020) (Figure 5A). The high-risk score group was characterized by the activation of basal differentiation, epithelial–mesenchymal transition differentiation, immune differentiation, interferon response, and so on (Figure 5A) and might be more sensitive to neoadjuvant chemotherapy (NAC) and immune therapy. On the contrary, the low-risk score group represented the luminal subtypes and was characterized by urothelial differentiation, Ta pathway, and luminal differentiation (Figure 5A). Importantly, the risk score’s predictive accuracy for molecular subtypes was extremely high, with a majority of ROCs being more than 0.90 (Figure 5B). The basal subtype has the features of more immune cell infiltration and higher response rates to immunotherapy, whereas the luminal subtype has the opposite features (Kamoun et al., 2020). The ability of TNF family–based risk score stratifying molecular subtypes of BLCA-revalidated risk score could predict TIME and ICB response from the aspect of molecular subtypes.

### Validation of the TNF Family–Based Risk Score Roles in the Xiangya Cohort

**FIGURE 6:**
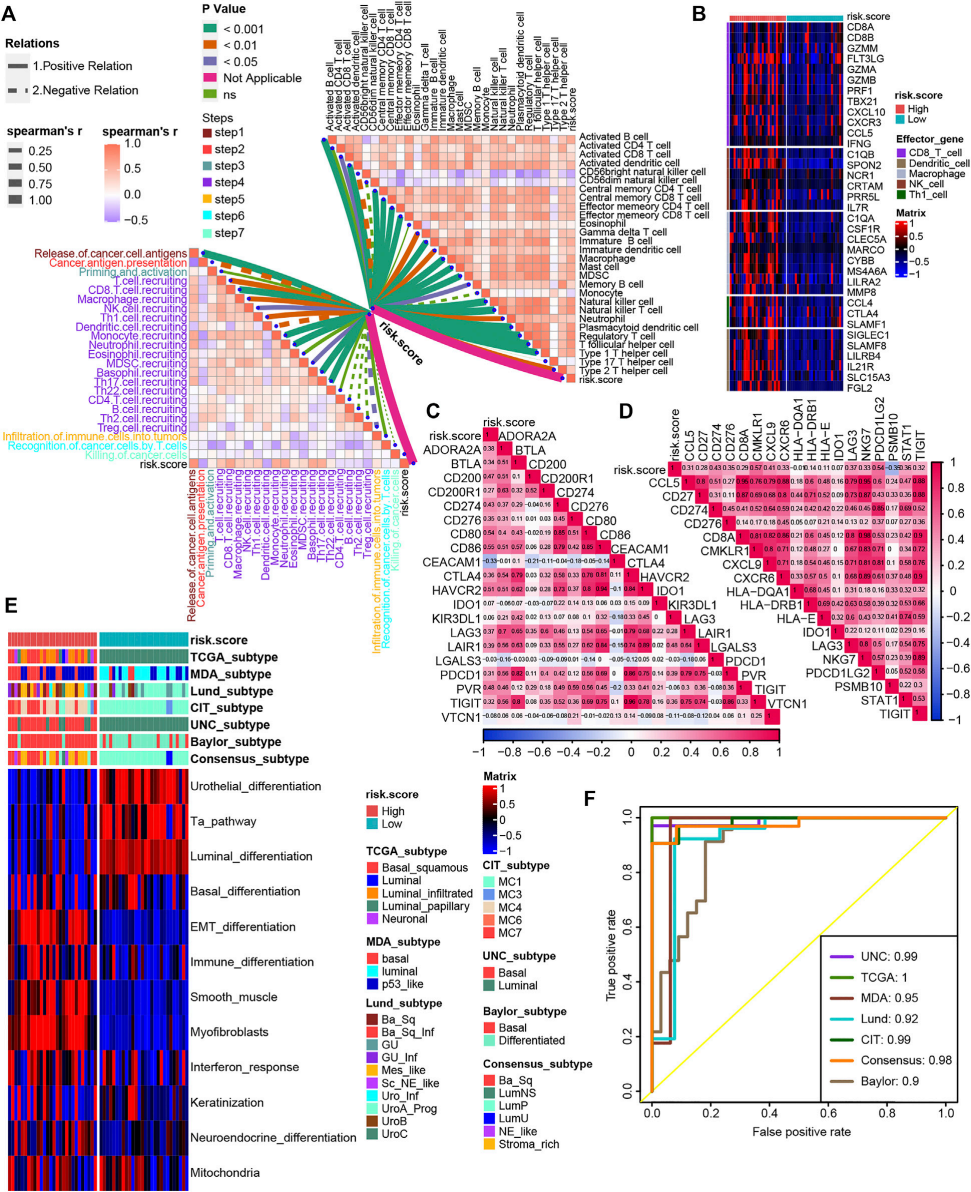
Validation of the TNF family–based risk score roles in the Xiangya cohort. **(A)** The association between TNF-based risk score, cancer-immunity cycles **(left)**, and immune cells in the TME **(right)** in the Xiangya cohort. The different types of lines represent the positive or negative relations. The different colors of the lines represent the *p* values of the relations, and the thickness of the lines represents the strength of the relations. **(B)** The different expression patterns of effector genes of immune cells between different TNF-based risk score groups in the Xiangya cohort. **(C**,**D)** The association between TNF-based risk score and ICI genes and TIS genes, respectively, in the Xiangya cohort. **(E)** The heat map of different TNF-based risk score groups, seven molecular subtype classifications, and bladder cancer–associated signatures in Xiangya cohort. Activated or inhibited pathways are marked as red or blue, respectively. **(F)** AUC plot of the risk score for predicting seven molecular subtype classifications in the Xiangya cohort.

We validated the relationship between TNF family–based risk score and TME and molecular subtypes in the Xiangya cohort. As expected, the TNF-based risk score was positively correlated with most steps of cancer-immunity cycles and immune cells in the TME in the Xiangya cohort (Figure 6A, Supplementary Table S11). For the effector genes of immune cells, patients with high-risk score expressed them higher than the low-risk score group (Figure 6B). Moreover, the risk score in the Xiangya cohort was also positively correlated with most of the ICI genes and TIS genes (Figures 6C, D, Supplementary Table S12). For molecular subtypes of BLCA, the patients with a high-risk score represented basal subtypes, whereas patients with a low-risk score represented luminal subtypes in the Xiangya cohort (Figure 6E). All ROCs for molecular subtypes were more than 0.90 (Figure 6F).

## Discussion

Our study was the first comprehensive investigation of the expression patterns and clinical and immunological roles of TNF family members in BLCA. We developed TNF-based patterns and correlated these patterns with prognosis and immune cell infiltration. In order to conduct a personalized evaluation of the role of TNF family members in BLCA, we have also developed and validated a TNF family–based risk score, which could be a robust tool for predicting prognosis. We further explored the relationship between the risk score and immune phenotypes and immunotherapy response of BLCA based on the specific role of TNF family members in TME. Moreover, TNF-based risk score could accurately stratify BLCA patients into basal and luminal subtypes, which reclarified the potential role for predicting the immune phenotypes and immunotherapy response from the aspect of molecular subtypes.

TNF family members, including 19 TNFSF ligands and 29 TNFRSF receptors, have a comprehensive shared ligand and receptor usage system. This system makes the TNF family members vital regulators of immunity through either coinhibited or costimulated ways (Dostert et al., 2019). TNFRSF6 can mediate the process of removing activated effector T cells, preventing immune damage by an over-long immune response (Dostert et al., 2019). Moreover, TNFRSF6 is essential for the process of cytotoxic T and NK cells killing tumor cells (Berke, 1995), whereas in its ligand, the expression of TNFSF6 in carcinoma cells could eliminate the infiltration of T cells and promote tumor proliferation and progression (Hahne et al., 1996; O'Connell et al., 1996). The higher expression of TNFRSF25 in activated T cells can promote T-cell proliferation and inflammatory cytokine production (Meylan et al., 2011; Ward-Kavanagh et al., 2016). TNFRSF4, as a costimulatory receptor, could be upregulated in CD8^+^ T cells, CD4^+^ T cells, and T_H_1 cells after antigen recognition (Willoughby et al., 2017). Interferon-γ could upregulate the expression of TNFSF10 in T cells, NK cells, and DCs, which could induce the death of numerous carcinoma cells (Pitti et al., 1996; Dostert et al., 2019). Zhang et al. conducted comprehensive analyses of TNF family members and correlated them with prognosis, immune phenotypes, and immunotherapy in lung adenocarcinoma (LUSC) (Zhang et al., 2020). In their study, they also developed a TNF-based risk score correlated with prognosis and immune cell infiltration in LUSC. However, their predictive accuracy remained unclear. In our study, we developed two risk scores using RSF and Cox proportional hazard regression analysis, respectively. We found that the risk score developed by RSF possessed higher predictive accuracy. Li et al. compared the RSF with other prediction models and found that RSF performed best with the highest AUC (Yang et al., 2020). The reasons could be that RSF could deal with regression and classification problems at the same time and accept dirty data. As far as we know, this is the first TNF-based risk score predicting the prognosis of BLCA.

ICB therapies, including programmed cell death-1, its ligand, and cytotoxic T-lymphocyte associated protein-4, have been approved for the treatment of multiple carcinoma types, such as advanced melanoma, renal cell carcinoma, non–small cell lung cancer, and BLCA (Reck et al., 2016; Motzer et al., 2018; Jenkins and Fisher, 2021). However, response rates to ICB therapies are approximately only 15% to 20% across different carcinomas (Osipov et al., 2019). TME, consisting of carcinoma cells, immune cells, stromal cells, and extracellular molecules, plays a key role in the efficacy of immunotherapy (Son et al., 2017). The TME can be divided into noninflamed (cold) and inflamed (hot) phenotypes based on the levels of T-cell infiltration and inflammatory cytokine (Duan et al., 2020). The inflamed phenotype of TME is reported to be more sensitive to ICB therapy as the preexisting T cells in the TME are a vital determinant for immunotherapy response (Liu et al., 2020). Distinguishing the immune phenotypes of BLCA is a promising way for improving ICB response rates in BLCA. Many efforts are committing to developing a risk score for immune phenotypes (Wang et al., 2020; Liu et al., 2021), and this is the first TNF-based risk score for predicting immune phenotypes in BLCA. We not only correlated the risk score with TME immune cell infiltration in pubic databases, but also validated its role in TME using the RNA-seq cohort developed by our hospital (Xiangya cohort), which made our risk score a more robust and reliable tool for predicting immune phenotypes. Moreover, we found that the TNF-based risk score could predict the ICB response rate using 21 gene signatures closely associated with clinical response to ICB therapy from the study by Mariathasan et al. (Mariathasan et al., 2018).

BLCA is a biologically heterogeneous disease and has different clinical outcomes and responses to therapies. Besides the conventional staging system using clinicopathological features, more and more studies are focusing on dividing BLCA into molecular subtypes using gene expression profiling and unsupervised analyses (Choi et al., 2014; Kamoun et al., 2020). Until now, there are seven reported BLCA molecular classifications, including TCGA, MDA, Lund, CIT, UNC, Baylor, and consensus subtypes (Kamoun et al., 2020). Generally, the basal subtype of BLCA patients possesses more immune cell infiltration and could be more sensitive to immunotherapy and NAC. However, the different numbers, sizes, and names of molecular subtypes and the complicated detected methods impeded the clinical application of all these seven classifications. The TNF-based risk score developed by us could distinguish basal and luminal subtypes of BLCA with high predictive accuracy, which could advance the clinical application of the molecular subtypes. Interestingly, although more immune cells are infiltrating into TME in the basal subtype of patients, this subtype is associated with poorer survival outcomes (Choi et al., 2014; Warrick et al., 2019). This could explain why the TNF risk score represented an inflamed phenotype of BLCA and was associated with poorer survival outcomes.

It should be acknowledged that there are some limitations. First, although we validated our risk score in two public databases and our Xiangya cohort, all these cohorts were retrospective cohorts, and prospective cohorts are still needed for further validation. Second, immune profiles in the TME are complicated and regulated by numerous factors. We did not validate the TNF family members’ role in TME *in vivo* and *in vitro*. Third, our training cohort (TCGA) was developed using RNA-seq, whereas two of our validation cohorts (GSE32894 and GSE13507) were developed using microarray. We could not correct for the batch effects. However, our risk score could be robustly validated in validation cohort regardless of batch effects caused by different sequencing platforms, indicating that our risk score was reliable.

## Conclusion

We developed and validated a robust TNF-based risk score, which could predict prognostic outcomes, TME, and molecular subtypes of BLCA. However, the value of risk score predicting the efficacy of immunotherapy needs further research.

## Data Availability

The datasets presented in this study can be found in online repositories. The names of the repository/repositories and accession number(s) can be found in the article/Supplementary Material.

## Glossary

TNF: tumor necrosis factor
BLCA: bladder carcinoma
TCGA: the cancer genome atlas
GEO: gene expression omnibus
LASSO: least absolute shrinkage and selection operator
RSF: random survival forest
TME: tumor microenvironment
DEG: differentially expressed gene
MIBC: muscle-invasive bladder carcinoma
NMIBC: non–muscle-invasive bladder carcinoma
OS: overall survival
FDA: US food and drug administration
ICI: immune checkpoint inhibitor
TNFRSF: TNF receptor
TNFSF: TNF ligand
RNA-seq: RNA-sequencing
FPKM: fragments per kilobase per million mapped fragments
GDC: Genomic Data Commons
TPM: transcripts per kilobase million
TIME: tumor immune microenvironment
TIS: T cell–associated inflammatory signature
TIP: tracking tumor immunophenotype
ssGSEA: single-sample gene set enrichment analysis
DC: dendritic cell
NK cell: natural killer cell
**T**_**H**_ **1 cell**: Type 1 T helper cell
GSVA: gene set variation analysis
ICB: immune checkpoint blockade
FC: fold change
GO: Gene Ontology
KEGG: Kyoto Encyclopedia of Genes and Genomes
K-M: Kaplan–Meier
ROC: receiver operating characteristic
MDA: MDAnderson Cancer Center
CIT: Cartes d’Identité des Tumeurs-Curie
UNC: University of North Carolina
NAC: neoadjuvant chemotherapy
LUSC: lung adenocarcinoma
