# Supplementary figures and images for: TNF Family–Based Signature Predicts Prognosis, Tumor Microenvironment, and Molecular Subtypes in Bladder Carcinoma
Source: Front Cell Dev Biol. 2022 Jan 31;9:800967. doi: 10.3389/fcell.2021.800967 (PMC8842074; doi:10.3389/fcell.2021.800967)

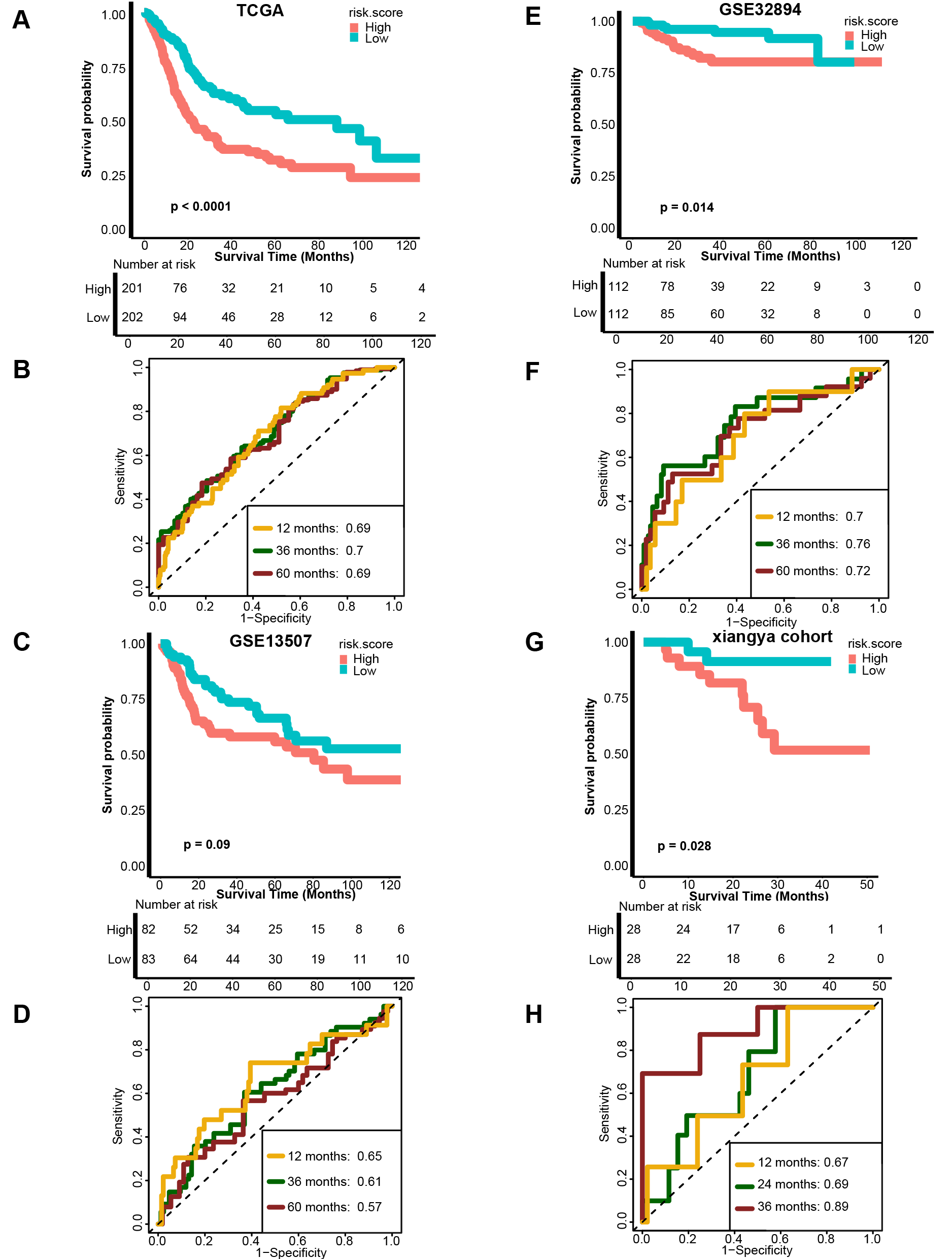

Supplement: Supplementary file 4 [file Image3.TIF]

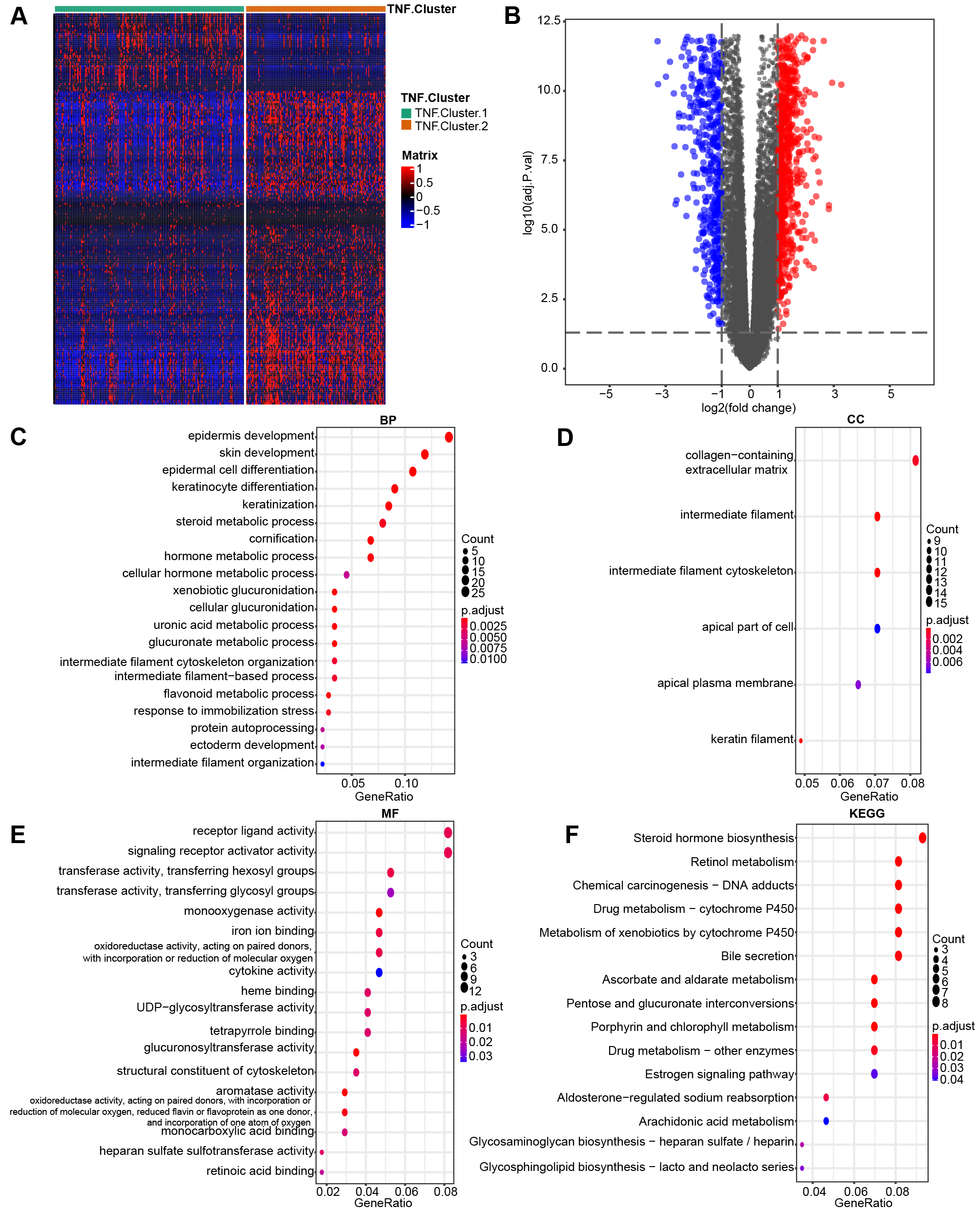

Supplement: Supplementary file 5 [file Image2.TIF]

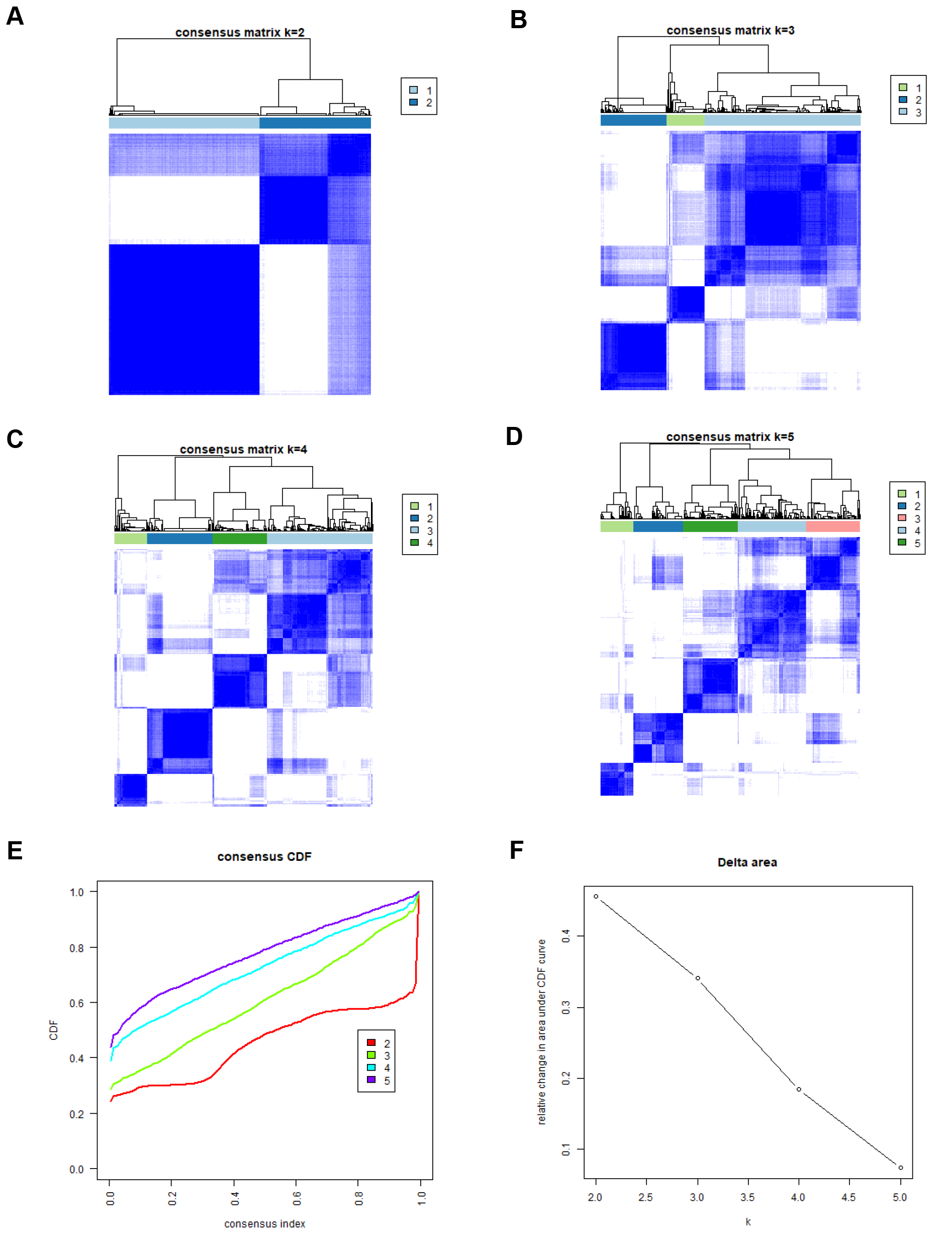

Supplement: Supplementary file 6 [file Image1.TIF]
